# Simultaneous Neutralization and Innate Immune Detection of a Replicating Virus by TRIM21

**DOI:** 10.1128/JVI.00647-13

**Published:** 2013-07

**Authors:** R. E. Watkinson, J. C. H. Tam, M. J. Vaysburd, L. C. James

**Affiliations:** Medical Research Council Laboratory of Molecular Biology, Division of Protein and Nucleic Acid Chemistry, Cambridge Biomedical Campus, Cambridge, United Kingdom

## Abstract

Tripartite motif-containing 21 (TRIM21) is a cytosolic immunoglobulin receptor that mediates antibody-dependent intracellular neutralization (ADIN). Here we show that TRIM21 potently inhibits the spreading infection of a replicating cytopathic virus and activates innate immunity. We used a quantitative PCR (qPCR)-based assay to measure *in vitro* replication of mouse adenovirus type 1 (MAV-1), a virus that causes dose-dependent hemorrhagic encephalitis in mice. Using this assay, we show that genetic ablation of TRIM21 or chemical inhibition of either the AAA ATPase p97/valosin-containing protein (VCP) or the proteasome results in a >1,000-fold increase in the relative level of infection in the presence of immune serum. Moreover, the TRIM21-mediated ability of antisera to block replication was a consistent feature of the humoral immune response in immunized mice. In the presence of immune sera and upon infection, TRIM21 also activates a proinflammatory response, resulting in secretion of tumor necrosis factor alpha (TNF-α) and interleukin-6 (IL-6). These results demonstrate that TRIM21 provides a potent block to spreading infection and induces an antiviral state.

## INTRODUCTION

The importance of antibodies in immune protection has long been recognized (1). Due to their collective broad binding repertoire, potential for affinity maturation, and multiple effector mechanisms, antibodies are able to act against the wide range of rapidly evolving pathogens from which the body is under attack. Until recently, antibodies were believed to exert their protective function exclusively in the extracellular environment (1). However, it has now been shown that antibodies are carried into cells by intracellular pathogens to which they are bound (2). Once in the cytosol, antibodies facilitate two processes: antibody-dependent intracellular neutralization (ADIN) and innate immune activation (3). Both mechanisms are mediated by tripartite motif-containing 21 (TRIM21), a universally expressed cytoplasmic antibody receptor, which binds with high affinity to the antibody Fc region via its PRYSPRY domain (4, 5). Upon recognition of antibody, TRIM21 initiates a degradation response involving both the segregase/unfoldase p97/valosin-containing protein (VCP) (6) and the proteasome (2). Together, these complexes mediate the rapid destruction of virus, resulting in efficient neutralization of infection. Concurrently with neutralization of virus, TRIM21 catalyzes the formation of free K63 ubiquitin chains via its RING domain, thereby activating NF-κB, AP-1, and IRF3/5/7 signaling pathways and leading to the production of proinflammatory cytokines (3).

Previous studies of TRIM21 activity have been conducted *in vitro*, primarily using a human adenovirus 5 vector under conditions where the virus cannot replicate. Thus, while TRIM21 may provide crucial antiviral protection, its role in preventing a spreading infection remained to be evaluated. Here we investigate the antiviral activity of TRIM21 during infection of a replication-competent wild-type (WT) virus, mouse adenovirus type 1 (MAV-1). MAV-1 is a nonenveloped icosahedral DNA virus (7), first isolated in 1960 (8), which causes dose-dependent hemorrhagic encephalitis in susceptible strains of mice (9). It is structurally (10) and genomically (11) similar to human adenovirus and provides an excellent model for the study of host and viral determinants of productive infection. Using MAV-1, we show that antibody-dependent TRIM21 activity provides a highly potent block to spreading viral infection.

## MATERIALS AND METHODS

### Cell lines and viruses.

Mouse embryonic fibroblasts (MEFs) were derived as described previously (13) from wild-type C57BL/6 (WT) or TRIM21 knockout (K21) embryos in which TRIM21 is replaced by enhanced green fluorescent protein (eGFP) (14). Cells were grown in Dulbecco's modified Eagle's medium (DMEM) supplemented with 100 U/ml penicillin, 100 μg/ml streptomycin, and 10% fetal calf serum at 37°C in a 5% CO_2_ atmosphere. MAV-1 (ATCC) was prepared by two rounds of CsCl centrifugation. MAV-1 antiserum was prepared from blood collected from C57BL/6 mice 72 days postinfection following 3 rounds of immunization with a sublethal dose of MAV-1. Immunizations were conducted in accordance with the 19.b.7 moderate severity limit protocol and Home Office Animals (Scientific Procedures) Act (1986).

### TCID_50_ assay.

WT and K21 MEF cells were seeded at 1 × 10^4^ cells per well in 96-well plates and allowed to adhere overnight. MAV-1 was diluted in serum-free DMEM over a range of 1:200 to 1:1 × 10^10^ per ml, and 100 μl was applied per well. After 14 days, cell viability was determined using PrestoBlue reagent (Invitrogen) according to the manufacturer's protocol. Values for the 50% tissue culture infective doses (TCID_50_) were calculated according to the Reed and Muench method (12).

### Neutralization by TCID_50_ assay.

Cells were seeded in 96-well plates at 1 × 10^4^ cells per well and allowed to adhere overnight. MAV-1 (dilution range from 1:10 to 1.6 × 10^6^) preincubated for 1 h with MAV-1 antiserum diluted 1:10,000 was applied to cells and incubated for 14 days. The apparent TCID_50_ in the presence of antiserum was calculated as described above.

### MAV-1 qPCR infection and neutralization assay.

Wild-type or TRIM21 knockout MEFs were plated at 6 × 10^4^ per well in 24-well plates and allowed to adhere overnight. MAV-1 was diluted in serum-free DMEM to the stated dose. A 200-μl volume of virus or virus preincubated with pooled immune serum (collected from MAV-1-immunized mice 72 days postinfection [dpi] and heat inactivated at 56°C for 1 h) was added to cells and allowed to adsorb for 4 h at 37°C. An 800-μl volume of DMEM with 1.25% serum was added, and cells were observed for 4 days unless otherwise stated. Cells were washed 3 times with phosphate-buffered saline (PBS), subjected to a trypsin procedure, and pelleted by centrifugation. Total DNA was purified using a QIAamp DNA minikit (Qiagen) in accordance with the manufacturer's protocol, and 100 ng was used per quantitative PCR (qPCR).

Neutralization assays in which N2,N4-dibenzylquinazoline-2,4-diamine (DBeQ) or MG132 inhibitors were used were conducted as described above except that cells were pretreated for 4 h with 10 μM DBeQ, 10 μM MG132, or a dimethyl sulfoxide (DMSO) equivalent in 150 μl serum-free DMEM prior to infection. Virus and antibody at a 4× concentration was then added in 50 μl so that the final volume during infection was 200 μl. At 4 h postinfection, cells were washed 3 times with PBS and then incubated in 1 ml of DMEM with 1% serum for 4 days. Alpha interferon (IFN-α) was added at 2,000 U/ml at the time of cell seeding.

### qPCR.

qPCRs were conducted using an ABI StepOne Plus Real Time PCR machine. The reaction volume was 20 μl, comprising 10 μl 2× TaqMan universal gene expression master mixes (ABI), 1 μl of 20× primer and probe mix (Eurofins) with the sequences 5′TTTTGTCCTGTGGCATTTGA (MAV-1 Hexon reverse), 5′GGCCAACACTACCGACACTT (MAV-1 Hexon forward), and 5′CATTCCAGCCAACTTATGGCTCGGC(MAV-1 Hexon 6-carboxyfluorescein/6-carboxytetramethylrhodamine [FAM/TAMRA] probe), and 100 ng purified genomic DNA diluted to 9 μl of total volume.

### Enzyme-linked immunosorbent assay (ELISA) for anti-MAV-1 IgG.

StreptaWell plates (Roche) were coated with biotinylated MAV-1 diluted 1:2,000 in PBS for 45 min and blocked in PBS–2% milk–0.05% Tween 20. Each serum was diluted 1:5,000 and 1:10,000 and incubated for 1 h at room temperature. IgG was detected using anti-mouse IgG-horseradish peroxidase (HRP) diluted 1:1,000 and TMB solution (eBioscience) with acid stop. Plates were read using a SpectraMax 340 plate reader at 450 nm and 650 nm.

### Cytokine assays.

WT and K21 MEF cells were plated at 2.5 × 10^3^ cells/well in a 96-well plate. The following day, 5 × 10^4^ TCID_50_ MAV-1 was mixed with immune serum diluted 1:3 (or PBS substituted for each component) in a total volume of 10 μl, incubated at room temperature for 1 h, and then added to each well. At 96 h postinfection, the supernatant was collected and applied to interleukin-6 (IL-6) and tumor necrosis factor alpha (TNF-α) ELISA kits (Life Technologies) according to the manufacturer's protocols and read as described above.

## RESULTS

### TRIM21 mediates neutralization of MAV-1 by antiserum.

**Fig 1:**
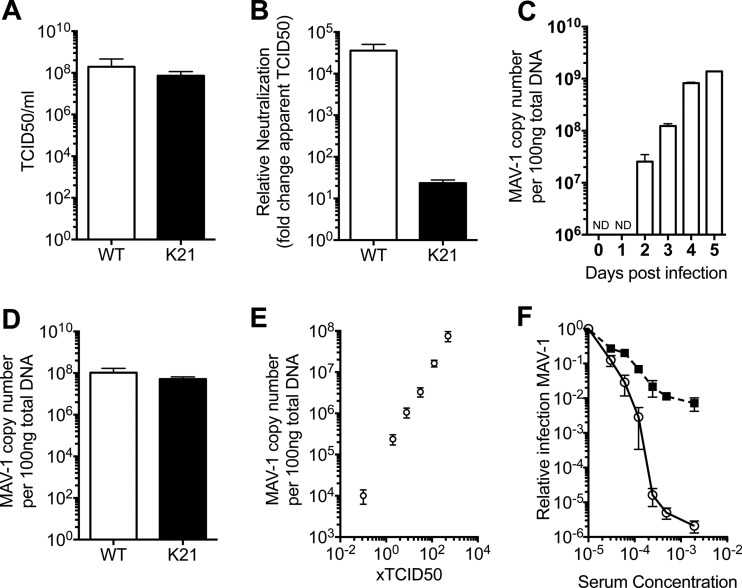
TRIM21 mediates *in vitro* neutralization of MAV-1 by antisera raised in mice as measured by TCID_50_ and qPCR. (A) MAV-1 TCID_50_/ml in MEF cells isolated from wild-type (WT, white bar) and TRIM21^−/−^ (K21, black bar) C57BL/6 mice. Error bars show standard errors of the means of the results from 3 experiments. (B) Fold change in observed MAV-1 TCID_50_ value upon addition of MAV-1 antiserum diluted 1:10,000, all determined by endpoint dilution assay in WT (white bar) and K21 (black bar) MEF cells. Error bars show standard errors of the means of the results from 3 experiments. (C) Viral replication following MAV-1 infection of WT MEFs measured between days 0 and 5 postinfection (ND, not detected). Error bars show standard errors of the means of the results from 3 technical replicates. (D) MAV-1 load 4 days postinfection with 500× TCID_50_ in WT (white bar) and K21 (black bar) MEF cells. Error bars show standard errors of the means of the results from 3 technical replicates. (E) Viral load in WT (white bar) and K21 (black bar) cells 4 postchallenge with MAV-1, as measured by qPCR for viral DNA. Error bars show standard errors of the means of the results from 3 technical replicates. (F) Relative MAV-1 infection levels in WT (white circles) and K21 (black squares) MEFs in the presence of immune mouse antisera. Viral loads were measured by qPCR for viral DNA 4 days postinfection. Error bars show standard errors of the means of the results from 3 technical replicates.

In order to determine whether genetic ablation of TRIM21 affects MAV-1 infection and replication, we infected mouse embryonic fibroblast (MEF) cells derived from C57BL/6 wild-type (WT) and TRIM21^−/−^ (K21). Virus was allowed to replicate for 14 days, after which cells were examined for cytopathic effect and the titer was calculated using the TCID_50_ method. We observed no differences between the WT and K21 MEF cells in viral titers (Fig. 1A), indicating that TRIM21 deletion has no significant impact on MAV-1 replication. We then assessed the contribution of TRIM21 in neutralization of MAV-1 by challenging WT and K21 MEF cells with MAV-1 in the presence of antiviral antisera diluted 1:10,000. We observed neutralization in both cell types; however, immune serum was 100-fold more protective in WT cells than in cells lacking TRIM21 (K21) (Fig. 1B). This demonstrates that TRIM21 ADIN provides substantial protection against a replicating virus and represents a significant component of neutralization by antiserum.

In order to investigate the relative importance of TRIM21 under different neutralizing conditions, we developed a qPCR-based assay using primers against hexon, the primary capsid component of MAV-1. Using this approach, we tested MAV-1 infection of WT and K21 MEFs. Replication can be reliably measured from 2 days postinfection, and viral load increases proportionally until day 5, after which the cytopathic effect of the virus precludes further measurement (Fig. 1B). At 4 days postinfection, viral load can be reliably determined following initial infection with virus at between 0.01× TCID_50_ and 500× TCID_50_ (Fig. 1C). Linear regression over this range reveals a strong linear relationship (*R*^2^ = 0.834) between the dose of infection and the measured viral titer, confirming the suitability of this as a fast and accurate alternative to the TCID_50_ assay for quantifying viral infection and replication.

Using this qPCR method, we confirmed that there was no measurable difference between WT and K21 MEF cells in viral replication (Fig. 1D). We then measured MAV-1 neutralization in WT and K21 cells by MAV-1 immune serum at a range of dilutions from nonneutralizing through to the persistent fraction. The persistent fraction is determined by the level of infection that remains at saturating concentrations of neutralizing antibody (13). We observed greater neutralization in WT cells than K21 cells at all dilutions of antiserum. Under conditions of maximal neutralization, MAV-1 antiserum was over 2,000-fold (*P* < 0.01) more potent in WT than K21 MEFs (Fig. 1D). These data are in agreement with results obtained by the TCID_50_ assay, although the increased dynamic range of the qPCR assay allows a greater fold change to be observed between cell lines.

**Fig 2:**
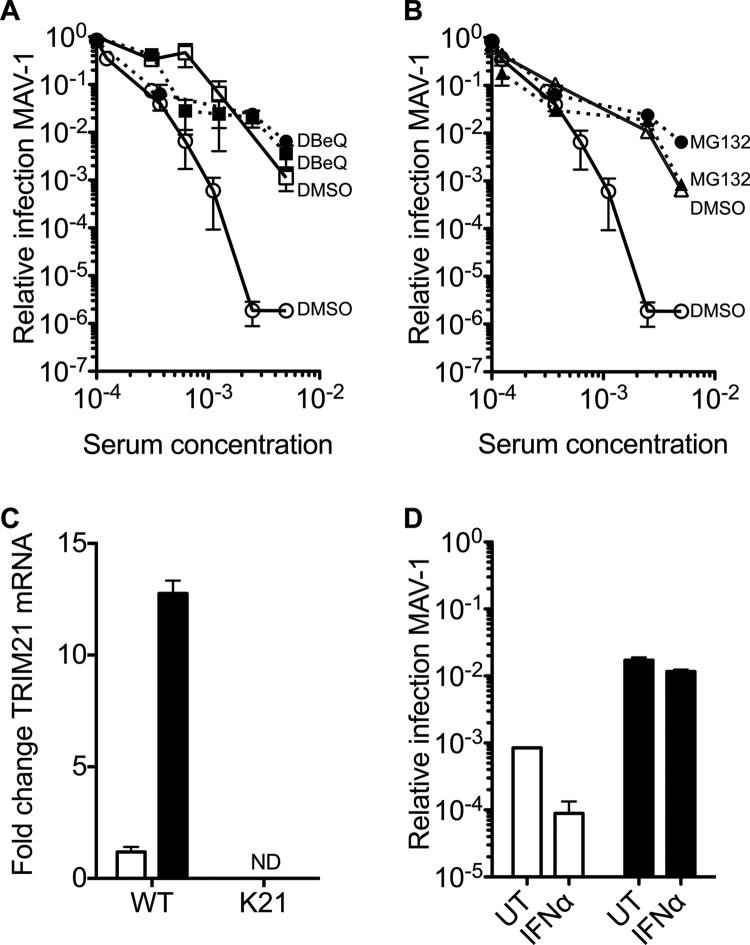
TRIM21-mediated neutralization is ablated by inhibition of VCP or the proteasome and is increased by stimulation with IFN-α. (A) Relative MAV-1 infection levels in WT MEFs treated with DMSO (white circles) or DBeQ (white squares) and K21 MEFs treated with DMSO (black circles) or DBeQ (black squares) in the presence of immune mouse antisera. (B) Relative MAV-1 infection levels in WT MEFs treated with DMSO (white circles) or MG132 (white triangles) and K21 MEFs treated with DMSO (black circles) or MG132 (black triangles) in the presence of immune mouse antisera. (C) Fold change of TRIM21 transcript levels compared to untreated-cell levels upon overnight infection with 500× TCID_50_ MAV-1 (white bar) or treatment with IFN-α (black bar) in WT or K21 MEF cells (ND, not detected). (D) Neutralization of MAV-1 by antisera in WT (white bars) and K21 (black bars) MEF cells either under unstimulated (UT) conditions or following pretreatment with IFN-α.

Classic neutralization (e.g., entry blocking) is primarily dependent upon antibody concentration, whereas ADIN requires functional TRIM21 and both proteasome and VCP activity (2, 6). Pretreatment of cells with MG132, a proteasome inhibitor, or DBeQ, a reversible inhibitor of VCP (15), reversed neutralization in WT cells to levels comparable to that in K21 cells (Fig. 2A and B). Pretreatment of WT or K21 cells with either inhibitor had no impact on MAV-1 infection in the absence of antisera (data not shown). Similarly, neither inhibitor significantly affected neutralization of MAV-1 by antiserum in K21 cells (Fig. 2A and B). Overnight infection with 500× TCID_50_ MAV-1 did not affect TRIM21 mRNA transcript levels, but pretreatment with IFN-α upregulated TRIM21 gene expression in WT cells (Fig. 2C), while TRIM21 mRNA remained nondetectable in K21 cells. This IFN-α treatment enhanced neutralization in WT cells approximately 10-fold but had little effect on K21 cells (Fig. 2D). These results confirm that significant MAV-1 neutralization is mediated postentry via ADIN and can be enhanced by TRIM21 upregulation.

### TRIM21 dependence is a common feature of antisera.

**Fig 3:**
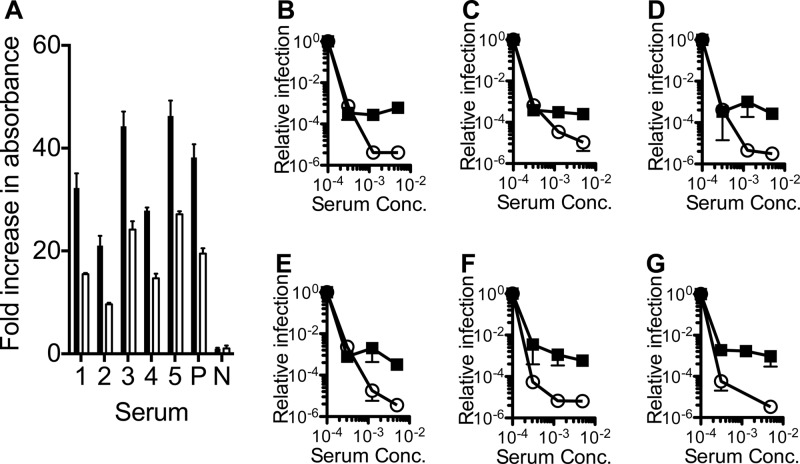
TRIM21 dependence of MAV-1 neutralization is a common feature of antisera raised in different individual mice. (A) Levels of specific anti-MAV-1 IgG detected in sera from immunized mice (1 to 5) and the pooled immune serum (P) normalized to absorbance from naive serum (N) from an uninfected mouse. Sera were diluted 1:5,000 (black bars) and 1:10,000 (white bars). (B to F) Relative MAV-1 infection levels in WT (white circles) and K21 (black squares) MEF cells in the presence of antisera from 5 individual mice. (G) Relative MAV-1 infection levels in WT (white circles) and K21 (black squares) MEF cells in the presence of antisera pooled from 30 mice.

Next, we investigated whether the importance of ADIN differs between antisera produced by different animals challenged with the same virus. First, we confirmed that all mouse sera tested had a high concentration of MAV-1-specific IgG by ELISA against MAV-1 (Fig. 3A). The levels of neutralization mediated by 5 different immune sera and pooled serum from 30 mice in WT versus K21 cells (Fig. 3B to G) were then compared. There was very little variation in the relative contributions of ADIN to neutralization in the comparisons between the antisera of different animals. This suggests that significant TRIM21-dependent neutralization is a general feature of the humoral immune response to MAV-1.

### TRIM21 upregulates proinflammatory cytokines in MAV-1-infected cells.

**Fig 4:**
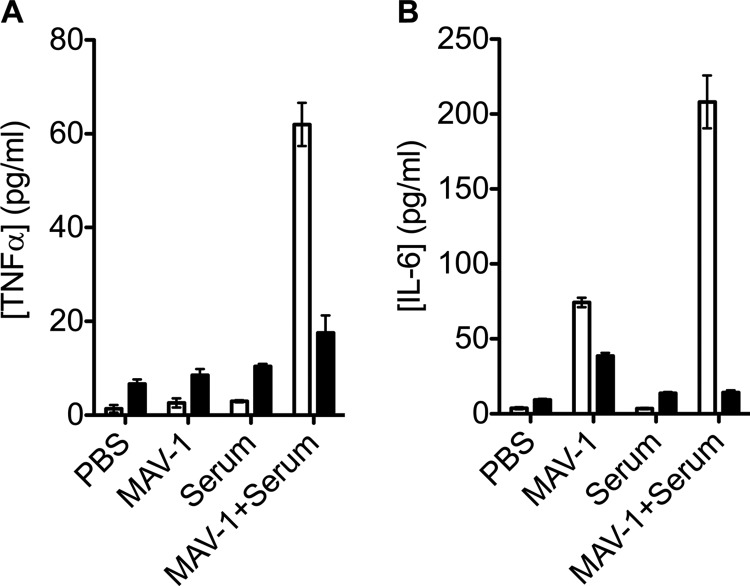
TRIM21 mediates antibody-dependent immune signaling. Data represent secretion of TNF-α (A) and IL-6 (B) upon treatment with MAV-1 and antiserum in WT (white bars) and K21 (black bars) MEF cells.

We have previously shown that the recognition of an antibody-coated virus in the cytosol by TRIM21 activates innate immunity and induces the secretion of proinflammatory cytokines (3). However, many viruses are adept at suppressing innate immune activation. For instance, adenovirus early region 1 proteins are implicated in repressing interferon-regulated genes (16) and the secretion of cytokine IL-6 (17). Whether adenoviral E1 proteins antagonize TRIM21 signaling is unknown, as previous experiments were carried out using virus lacking the E1 genes (3). To determine whether productive TRIM21 signaling occurs during normal wild-type virus replication, we measured tumor necrosis factor alpha (TNF-α) and interleukin-6 (IL-6) secretion by infected cells upon challenge with MAV-1. We compared responses between WT and K21 MEFs challenged with MAV-1 alone, MAV-1 antiserum alone, or MAV-1 preincubated with antiserum (Fig. 4). Incubation of either WT or K21 MEFs with either MAV-1 or antiserum alone did not significantly affect secretion of TNF-α. However, upon infection with MAV-1 preincubated with antiserum, secretion of TNF-α from WT cells increased by over 50-fold. This was strongly dependent on the presence of TRIM21, since there was an increase in secretion from K21 cells of less than 5-fold under the same conditions (Fig. 4A).

Infection of either WT or K21 MEFs led to an induction of IL-6 production (Fig. 4B). MAV-1 antiserum alone did not alter IL-6 secretion from resting levels. However, in WT cells, preincubation of MAV-1 with antiserum resulted in a significant increase in IL-6 production over and above that generated by infection with MAV-1 alone. Conversely, in K21 cells, preincubation of MAV-1 with antiserum resulted in a decrease in IL-6 secretion relative to that induced by MAV-1 alone. This was likely due to reduced infection levels as a result of entry neutralization. The fact that an increase in IL-6 is observed in WT cells in the presence of antisera suggests that TRIM21 promotes IL-6 production to levels that more than compensate for the reduction in titer that occurs as a result of neutralization.

## DISCUSSION

We have quantified the contribution of TRIM21 in neutralization of a replicating virus. We find that a significant proportion of MAV-1 neutralization mediated by specific IgG antiserum is dependent on the ADIN effector mechanism, since it requires TRIM21 (Fig. 1D), VCP enzymatic function (Fig. 2A), and proteasome activity (Fig. 2B). Furthermore, by testing antisera collected from 5 different immunized mice, we have confirmed that the dependence of MAV-1 antiserum neutralization on ADIN is a general feature of the humoral response to this virus (Fig. 3A and B). The relative importance of ADIN versus entry blocking in the neutralization of different viruses is likely to vary. However, viruses are thought to contain immunodominant epitopes that can bias the polyclonal response toward non-entry-blocking antibodies (18, 19). This may allow viruses to minimize the generation of antibodies against critical epitopes, such as those required for viral entry (20). This strategy may in turn have contributed to host evolution of ADIN.

We have also shown that the neutralization potency of antiserum can be increased in a TRIM21-dependent manner by pretreating target cells with IFN-α (Fig. 2C). This result is in good agreement with the reported effect of IFN-α modulation of TRIM21 gene expression on the efficiency of ADIN (13). It is of interest that addition of exogenous IFN-α has a substantial effect on neutralization in the context of infection by a replicating virus, which would be expected to activate immune signaling and therefore independently increase IFN-α and TRIM21 production. Small molecules capable of selective induction of TRIM21 expression may therefore have therapeutic value in enhancing the endogenous ADIN pathway as antiviral drugs of potentially broad specificity.

Finally, we have found that TRIM21 is important in inducing proinflammatory cytokine production upon infection with MAV-1. In the presence of MAV-1 and antiserum TRIM21 activates the secretion of TNF-α, which is known to be stimulated *in vivo* upon MAV-1 infection (21). IL-6 secretion is activated by virus alone, but only weakly, and is greatly enhanced by TRIM21 in the presence of antisera. Importantly, these studies have been carried out with wild-type replication-competent adenovirus that contains the early region 1 genes implicated in antagonism of immune activation and IL-6 secretion. This antagonism may explain why virus alone does not significantly induce IL-6 expression. Conversely, detection of incoming viral particles by TRIM21 prior to *de novo* viral synthesis may provide the cell with an early warning system that is harder to antagonize.
